# Changes in the Electrophysical Parameters of Nanomodified Elastomers Caused by Electric Current’s Passage

**DOI:** 10.3390/polym15010249

**Published:** 2023-01-03

**Authors:** Alexandr V. Shchegolkov, Aleksei V. Shchegolkov, Natalia V. Zemtsova, Yaroslav M. Stanishevskiy, Alexandre A. Vetcher

**Affiliations:** 1 Institute of Technology of the Department of Technology and Methods of Nanoproducts Manufacturing, Tambov State Technical University, 392000 Tambov, Russia; 2 Department of Chemical Technology, Platov South-Russian State Polytechnic University (NPI), 132 Enlightenment Str., 346428 Novocherkassk, Russia; 3 Department of Technique and Technology for Obtaining Nanoproducts, Tambov State Technical University, 106 Sovetskaya St., 392000 Tambov, Russia; 4 Institute of Biochemical Technology and Nanotechnology (IBTN), Peoples’ Friendship University of Russia (RUDN), 6 Miklukho-Maklaya St., 117198 Moscow, Russia; 5 Complementary and Integrative Health Clinic of Dr. Shishonin, 5 Yasnogorskaya St., 117588 Moscow, Russia

**Keywords:** MWCNTs, catalyst, polyurethane, organosilicon compound, modification, degradation, thermal field

## Abstract

The development of reliable and effective functional materials that can be used in various technological fields and environmental conditions is one of the goals of modern nanotechnology. Heating elements’ manufacturing requires understanding the laws of heat transfer under conditions of different supply voltages, as this expands the possibilities of such materials’ application. Elastomers based on silicon-organic compounds and polyurethane modified with multi-walled carbon nanotubes (MWCNTs) were studied at various concentrations of Ni/MgO or Co-Mo/MgO and voltages (220, 250, and 300 V). It was found that an increase in voltage from 220 to 300 V leads to an initial increase in specific power on one-third followed by a subsequent decrease in a specific power when switched on again to 220 V (for −40 °C) of up to ~44%. In turn, for a polyurethane matrix, an increase in voltage to 300 V leads to an initial peak power value of ~15% and a decrease in power when switched on again by 220 V (for −40 °C) to ~36% (Ni/MgO -MWCNT). The conducted studies have shown that the use of a polyurethane matrix reduces power degradation (associated with voltage surges above 220 V) by 2.59% for Ni/MgO–based MWCNT and by 10.42% for Co-Mo/MgO. This is due to the better heat resistance of polyurethane and the structural features of the polymer and the MWCNT. The current studies allow us to take the next step in the development of functional materials for electric heating and demonstrate the safety of using heaters at a higher voltage of up to 300 V, which does not lead to their ignition, but only causes changes in electrophysical parameters.

## 1. Introduction

The recent call for complex technological objects, both in industry and in the domestic sphere, has led to the formation of more effective concepts and strategies in the field of materials science, namely the creation of smart materials [1]. Smart materials can provide the implementation of various functional tasks, concerning their application for electric drive systems—materials with controlled deformation (electroactive elastomers) [2,3,4] and strain gauge [5], as well as electric heating in systems to combat ice.

On an industrial scale, it turned out to be effective to use polymer matrices with conductive dispersed additives. Variations between the properties of elastomer matrices [6] and conductive disperse additives make it possible to form a wide range of materials with various properties [7]. The dynamic development of electric vehicles is accompanied by new types of tasks that affect the improvement of microclimate technologies [8]. In order to increase comfort, the use of electric heating can affect various areas of the vehicle [9]. During the operation of electric vehicles, a significant part of the energy stored in the battery (up to 35% [10]) is consumed for heating, and, accordingly, the efficiency, reliability, and stability of heating systems are very important.

It is important to use heaters with the effect of maintaining the temperature regime due to the effect of self-regulation. This effect is realized in a PTCR (Positive Temperature Coefficient Resistor) [11]. Most electric heaters are based on the PTCR BaTiO_3_ effect [12]. The PTCR effect has built-in protection against overheating: the resistivity of the PTCR material increases by several orders of magnitude near the ferroelectric-paraelectric phase transition temperature (commonly referred to as the Curie temperature TC), which reduces the conductivity and, in turn, the heating current and power by orders of magnitude [11,12,13,14]. Accordingly, the PTCR heater does not heat up more than TC during normal operation. The Heyvang–Jonker model describes the PTCR effect and is based on temperature-dependent potential barriers at grain boundaries and their interaction with the ferroelectric domains of the fillers.

However, PTCR heaters are powered directly by the vehicle’s main batteries, which are between 500 V and 1 kV [15]. At these high voltages, PTCR materials show a degradation effect with increasing resistivity when voltage is applied. This increase, in turn, reduces the heating output and creates a serious problem for the application [15]. The fragility of ceramics and the difficulty of creating vibration-resistant electrical contacts should be noted.

For electric heaters, conducting networks in the structure of a dielectric matrix can be obtained using metal nanodots (Cr-nd, NiCr-nd, and Ni-nd) [16]. For heaters, polypropylene heaters can be used as a polymer matrix, and conductive structures are hybrid fillers [17] for opening the conductive network.

Composites based on CNTs and polymers can be an effective and technological solution in the field of electrical heating—due to their light weight, high design flexibility, and fast temperature response. Studies with the CNT/TPU heater [18] reported the possibility of maintaining the stability of the electrophysical properties under non-tensile deformations.

Consideration should be given to the possibility of conducting polymer nanocomposites with reversible dynamic bonds, as well as their energy activation for self-healing by heating when an electric current flows (Joule effect) [19]. Flexible composites based on silicone rubber (SR)/carbon fiber(CF)@polydopamine (PDA) (SR/CF@PDA) have good interfacial adhesion in the structure of the material and filler (due to the adhesive properties of PDA) [20], which ensures flexibility without compromising electrical properties. Polyurethane-coated carbon fiber (CF) can be added to the process for producing thermoplastic polymer composites with improved thermal and mechanical properties, as well as durability, as an effective reinforcing filler and an improved cross-linking process. Electron beam irradiation (EB) can be added [21]. In the framework of the technological process [21], composites based on polyamide 6 (PA) with different HC content from 1 to 10 wt.% were made by melt compounding and compression molding and then irradiated with various doses of EB.

Conductive composites based on organosilicon elastomers (silicones) [22] may have a functional feature associated with self-regulation of the heating temperature [23]. Studies related to the effect of MWCNTs on heat release in a flexible nanomodified elastomer are presented in the work and it was found that the heat release power can be represented in the form of regression equations, where there is a dependence of heat release on temperature, applied tensile strain and electrical heating [24]. In [25], the authors studied flexible heating elements based on silicone polymerized by a platinum catalyst and TiO_2_ with different morphologies (spherical and acicular), as well as an SnO_2_/Sb coating and carbon black. The presented heater had a dependence of resistivity on temperature and could operate on an alternating voltage of 220 V.

Conductive fillers, such as carbon nanotubes (CNTs), exhibit either metallic or semiconducting properties [26]. It should be noted that the morphological diversity of carbon nanomaterials, namely CNT and, in particular, multi-walled carbon nanotubes (MWNTs) with many concentric carbon shells or aligned single-walled nanotubes (SWNT), are complex composite conductors including many weakly bonded nanotubes, each of which has a different electronic structure and, consequently, electrical properties [27].

The morphology of MWNTs can be controlled using the method of selective removal of single carbon shells from the overall structure of MWNTs and SWNTs to adapt the properties of these composite nanotubes to any practical problems [26,27]. It is possible to remove the shells of MWNTs in stages and characterize different shells individually. The presented approach of the selective removal of layers makes it possible to convert MWNTs to either a metallic or semiconductor material, as well as directly solve the problem of transport with multiple shells [28].

When forming composites based on CNT, it is necessary to take into account the effect of structural distortions in CNT of the “armchair” type on electronic and electrical properties. Bending CNTs reduces their transfer function (electrical conductivity) in certain energy ranges and leads to an increase in electrical resistance. Electronic structure calculations show that these energy ranges contain localized states with significant σ-π hybridization as a result of the increase in curvature caused by bending. Twisting strongly affects the electronic structure of the CNT. In an ordinary metal chair (n,n) CNT, a band gap is formed, which first increases linearly with the angle of twist and then reaches a constant value. This saturation is associated with a structural transition to a flattened helical structure. The calculated values of the torsion energy and band gap are strongly affected by the possibility of structural relaxation in twisted structures [29].

The flow of current through the CNT can cause obvious destruction in the structure of the CNT. The mechanism of CNT destruction under the action of an electric voltage (potential difference) (a high current load causes local thinning of the nanotube in the case of MWCNT structures) is presented in [30]. The mechanism of destruction of SWCNT can be explained as follows: oxidation due to Joule heating occurring at the center of the line. In addition, the destruction of several samples of semiconductor and metal SWCNT samples occurred not only in the center but also on the cathode side of the line [31].

Unlike metallic wires, MWNTs do not break down in the continuous, accelerating manner that is characteristic of electromigration. Instead, they degrade due to a series of sharp surges of electric current of the same intensity. Degradation is associated with the successive destruction of individual nanotube shells following the geometry of the concentric shell of MWNTs. In addition, the initiation of this destruction is very sensitive to the oxidative action of air. In the air, destruction is initiated by oxidation at a certain power (current value), while in a vacuum, MWNTs can withstand much higher power (current) densities and reach the maximum current carrying capacity [32]. It was shown that the destruction of carbon nanotubes is based on a pulsating current flow process with pronounced peaks [33].

It was found that the thermal degradation of CNT composites as a result of direct current flow (E-heating) is much more serious than convection heating (C-heating). Increased mechanical properties of the CNT composite were observed after 40 h C-heating, while E-heating made the composites brittle such that the matrix was severely damaged at the same temperature/hours. Consequently, the electrical resistance of the CNT composite for E-heating was dramatically increased. To avoid thermal degradation and a sharp increase in durability, the E-heating temperature must be well below the temperature limit of the thermally stable polymer. In addition, the thermal degradation of the polymer matrix can be reduced by adding CNT, which in essence can be explained by the formation of a parallel resistor, by distributing the thermal concentration throughout the polymer matrix [34]. It is possible to improve the characteristics of CNT by using the technology of obtaining hybrids—graphene is of particular interest in this regard. Graphene–CNT hybrids (G/CNT) were formed by in situ Joule heating in a transmission electron microscope (TEM). The formation of the G/CNT structure occurs as a result of the sequential and spontaneous unfastening of the extreme wall of the MWCNT under uniform thermal etching and a voltage pulse of 0.2–1 V. The conductivity of the G/CNT hybrids shows a significant change (up to 38 times) after decorating with CdTe quantum dots [34,35].

It should be noted that the correlation between the electrical conductivity and the content of the conductive dispersed filler is important from the point of view of the general theory of percolation [36]. There are models explaining how the microstructure of electrically conductive polymer nanocomposites affects their temperature coefficient of resistance, which is crucial for Joule heating. There is also a relationship between morphology and local overheating, as well as impurities introduced by CNT, in terms of the resistance of composites to thermal decomposition [37].

To fully understand the formation of efficient conductive composites, it is necessary to combine studies of electrical conductivity measurement and modeling aspects of CNT/polymer composites obtained through the production of fused filament (FFF) and additive manufacturing (AM). Raw materials for CNT/polylactic acid (PLA) and CNT/high-density polyethylene (HDPE) filaments have been synthesized by controlled-feed CNT melt blending to create polymer nanocomposites. The electrical conductivity of 3D printed CNT/PLA and CNT/HDPE composites was measured under various CNT loads. Low percolation thresholds were obtained from measurement data as 0.23 vol.% and 0.18 vol.% CNT for CNT/PLA and CNT/HDPE nanocomposites, respectively. A two-parameter model of CNT agglomeration based on micromechanics makes it possible to predict the electrical conductivity of CNT/polymer composites [38].

The thermal degradation of PA11/MWCNT composites is also affected by the amount of MWNT used to fill the polymer matrix. MWCNTs were prepared by the catalytic chemical precipitation of methane over Co-Mo/MgO catalysts and then treated in 2.6 M nitric acid to remove catalyst residues. The PA11/MWCNT composite becomes more thermally stable as the mass concentration of MWNT increases to 1 wt.%. This is manifested in the fact that the composite containing 1 wt.%. MWNT has the best thermal stability and the decomposition temperature improves to around 20 °C. At a higher content of MWCNT (for example, 2 wt.%), the PA11/MWCNT composite is destroyed at a lower temperature, compared with nanocomposites with a lower content of MWCNT (for example, 0.5 and 1.0 wt.%), probably due to MWCNT aggregation in composites with higher MWCNT concentrations [39].

The presented analysis of composites for which there is a relationship between electrical resistance and temperature, which affects the Joule heating, or their behavior during thermal degradation (destruction of the polymer matrix or conductive structures predominates), differs from conventional monolithic heating materials (for example, metals that form oxides causing structural deterioration during the passage of an electric current).

The purpose of the current study was to evaluate the effect of high supply voltage on the process of degradation of conductive structures in nanomodified elastomers.

In accordance with it, the following tasks were set:

(1) Obtaining elastic organosilicon and polyurethane matrices of modified MWCNTs synthesized by chemical vapor deposition (CVD) technology;
(2) Study the electrically conductive nanomodified elastomers of heat release at a supply voltage in the range from 220 to 300 V.

## 2. Materials and Methods

For the MWCNTs’ synthesis, the CVD method was employed with Ni/MgO and/or Co-Mo/MgO as a catalyst for synthesis at a temperature of 700 °C. Silicon-organic compound Silagerm 8040 and polyurethane Silagerm 6030 (both from ELEMENT 14 LLC, Moscow, Russia) were employed as the polymer matrix of the elastomer(s).

Component (A)—an organosilicon compound—and MWCNTs were mixed on a WiseStir HT 120DX overhead mechanical stirrer (WiseStir Ltd., Seoul, Republic of Korea) at 200 rpm for 20 min. Then, the second component based on platinum (Pt) was introduced into the mixture, which provided polymerization (B), followed by stirring for 10 min at a temperature of 22 °C. The concentration of MWCNTs in the elastomer for Ni/MgO was 10 wt.% and Co-Mo/MgO was 3 wt.%. Geometric parameters were the length and width of the elastomer sample with MWCNTs of 10 cm and 7 cm (thickness of 2 mm).

**Figure 1:**
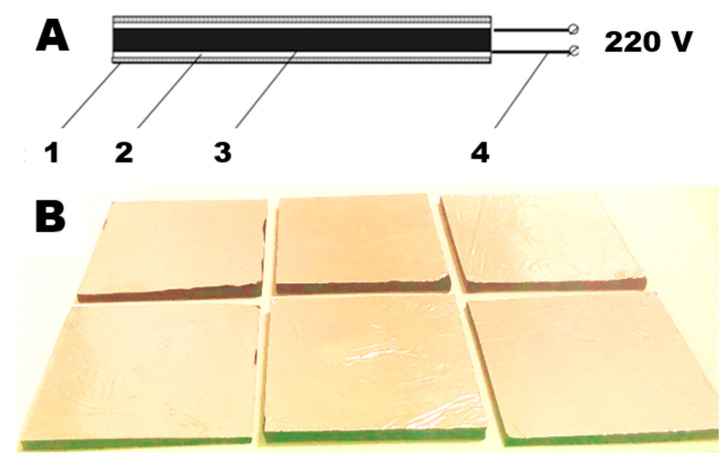
(**A**) Schematic diagram of a self-regulating constant current heater: 1—dielectric shell; 2—current collectors; 3—functional material of the heater; 4—connectors. (**B**) Heaters used in the current study.

Moisture was removed from MWCNTs before introduction into the elastomer in a SANYO CONVECTION OVEN MOV 210F drying oven (SANYO Ltd., Osaka, Japan) at 110 °C. The samples were obtained according to the technology described in [24,25] (Figure 1).

The obtained products are listed in Table 1.

A programmable power supply ATN 1351 (Eliks Ltd., Moscow, Russia) with a control range from 0 to 300 V was used as a power source. Employment of the ATN-1351-SW software makes it possible to evaluate the current consumed by the heater samples, which, taking into account the exposed voltage, allows you to find the value of the power consumption. According to the value of the found power, and taking into account the area of the heater sample, the specific power (W/m^2^) is found. Temperature tests of the heater were carried out in the range from −40 to +40 °C in the climate chamber “KTH-1000” (NPF Technology, LLC, St. Petersburg, Russia). To measure special volume conductivity a Tera Ohmmeter E6-13a (Punane-RET Ltd., Tallinn, Estonia) with a range of measuring electrical resistance up to 14 TΩ was employed.

### 2.1. Method for Studying the Temperature Field on the Surface of Samples of Nanomodified Elastomers

To study the temperature field, a Testo-875-1 thermal imager with a 32 × 23° optical lens (SE & Co. KGaA, Testo, Lenzkirch, Germany) was used with a distance of 10 cm from samples of nanomodified elastomers in a darkened room without exposure to sunlight. The temperature of nanomodified elastomers was measured with a two-channel thermometer, “Testo 992” (SE & Co. KGaA, Testo, Lenzkirch, Germany), while the surface temperature was determined. Based on the data obtained, a comparison was made with the temperature recorded by a thermal imager, after which the emissivity was selected and used for further measurements. The obtained thermal imaging images of composite samples with MWCNTs were processed using the IRSoft v4.9 SP1 program.

### 2.2. Structural Studies of MWCNTs and Elastomer’s Matrix

Structural studies of MWCNTs were carried out using the method of transmission electron microscopy. A small number of samples were made by contact with microscopic meshes with an adhesive composition. The studies were carried out from different places of the sample samples and on several samples in order to obtain better statistics about the samples under study. TEM and SEM studies were carried out using a Hitachi H-800 electron microscope with an accelerating voltage of up to 200 keV. An IR Fourier spectrophotometer FT 801 (Spectral range 21–1.8 µm) (LLC NPF Simeks, Novosibirsk, Russia) was used for the registration in the near and mid-IR range of the spectra of the original and modified with MWCNT elastomer matrix.

For TG and DSC studies, a NETZSCH STA 449F3 instrument (NETZSCH-Gerätebau GmbH, Selb, Germany) was used. Tests were carried out in the Ar-atmosphere.

## 3. Results

**Figure 2:**
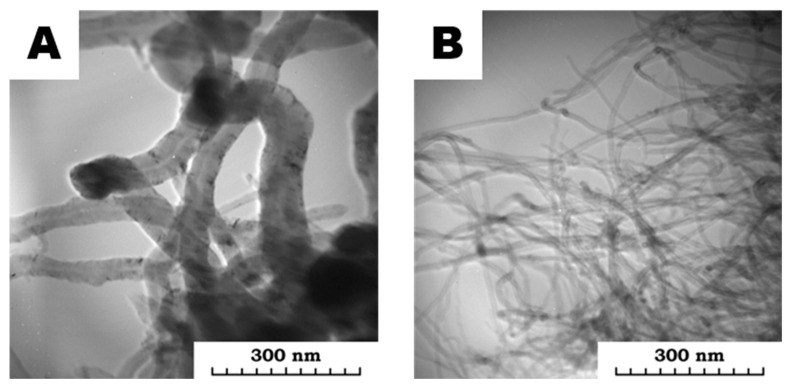
TEM of MWCNTs synthesized with (**A**) Ni/MgO and (**B**) Co-Mo/MgO catalysts.

Based on the graphical information presented on the TEM MWCNTs synthesized on Ni/MgO and Co-Mo/MgO catalysts (Figure 2), it can be concluded that there are “kink” deformations, which are characterized by different bending angles and spatial overlap, as well as inclusions of catalyst particles in the structure of an individual.

It should be noted that, as a rule, “kink” deformations occur in bent MWCNTs, as well as possible hybridization of bonds around these breaks (Figure 2). Inclusions of catalyst particles, as well as spatial defects, occur at various angles, including those significantly below 90°, and can significantly reduce the conductivity of MWCNTs, which leads to the formation of a current transition into thermal energy and local overheating zones.

**Figure 3:**
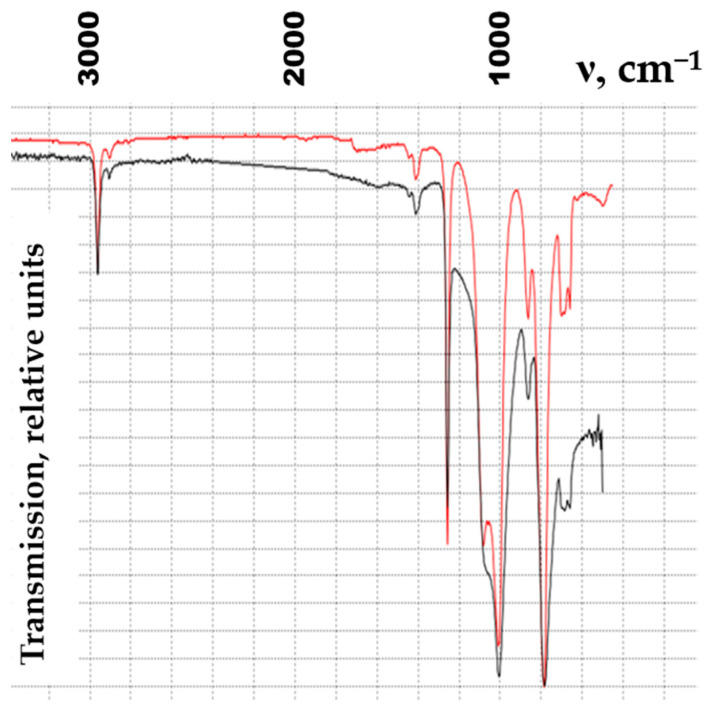
IR spectra of CO and NCO 1.

The addition of MWCNTs to an organosilicon matrix leads to a change in the molecular structure, which is associated with a change in the intensity of the peaks in the IR spectrogram. IR spectra of CO and NCO 1 are demonstrated in Figure 3.

**Figure 4:**
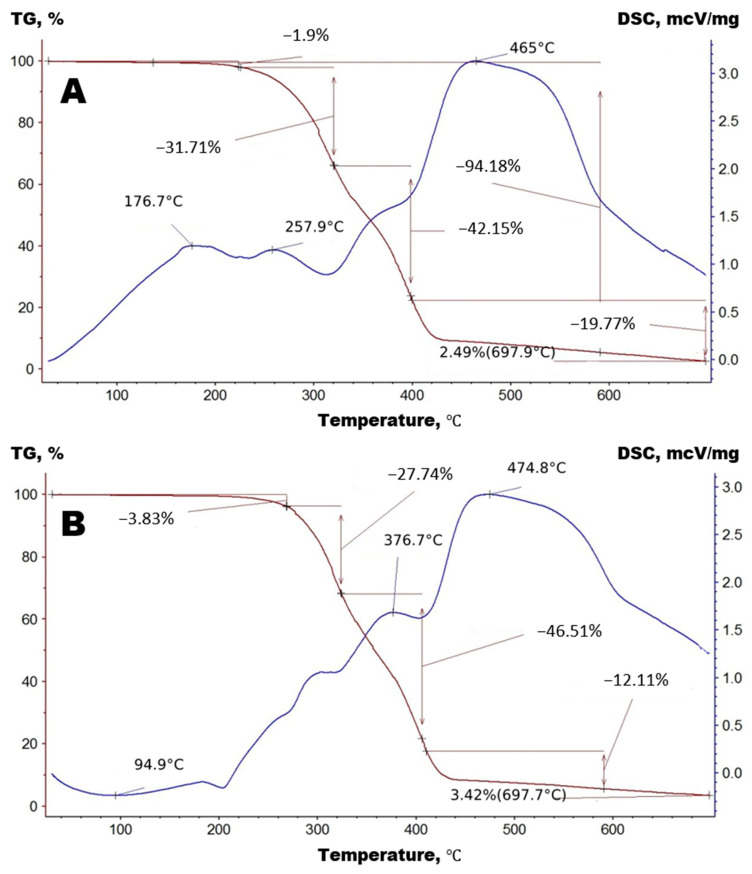
TG and DSC of (**A**) NPU 1; (**B**) NPU 2.

The test results of NPU 1 and NPU 2—TG and DSC—are shown in Figure 4.

The comparison of TG and DSC for NPU 1 and NPU 2 demonstrates differences caused by MWCNTs with different catalysts. The differences in the profiles of phase transitions are remarkable. The TG and DSC of the silicon-organic compound have already been reported [40].

**Figure 5:**
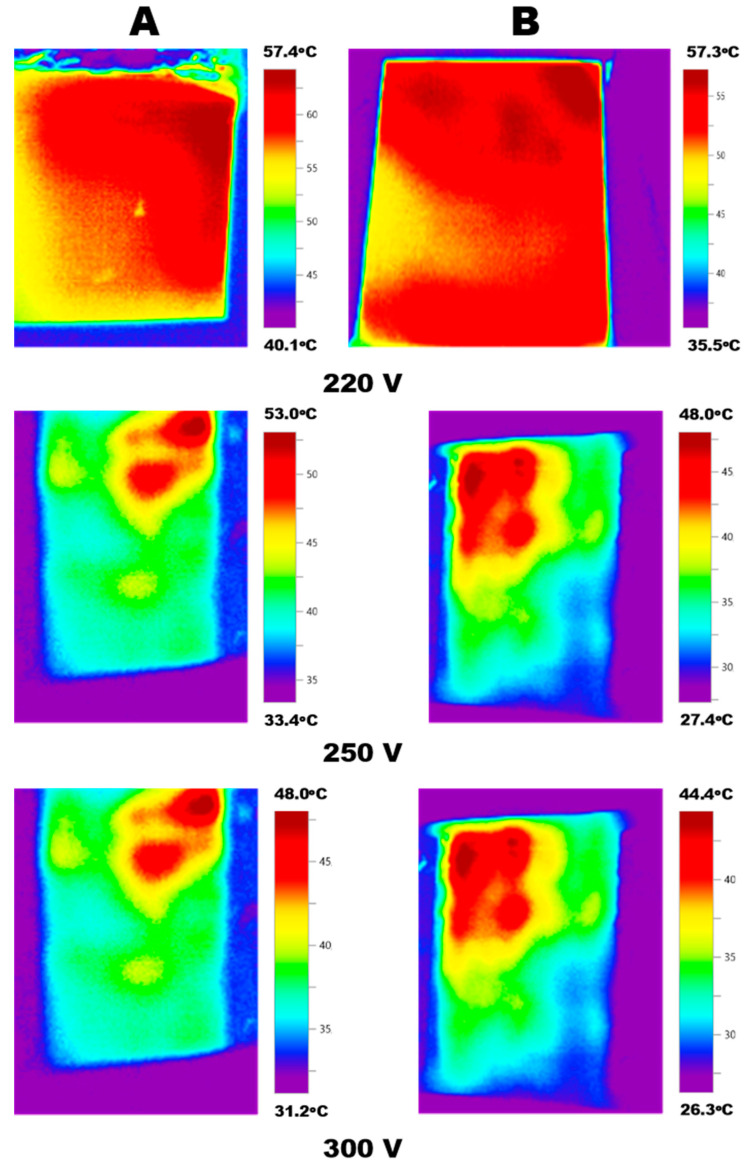
The comparison of the temperature fields for (**A**) NCO 1 and (**B**) NCO 2 at different voltages.

The temperature fields for the NCO 1 and NCO 2 are compared in Figure 5. An increments of voltage up to 250 V leads to the degradation of the conductive structures and the formation of a local inhomogeneity of the temperature field, which is expressed in partial heat release from the surface of the heater samples. Subsequent increments up to 300 V lead to the additional degradation of the conductive structures and the formation of a local inhomogeneity of the temperature field, which is expressed in partial heat release from the surface of the heater samples, as well as a decrease in the heat release temperature by 10 °C.

**Figure 6:**
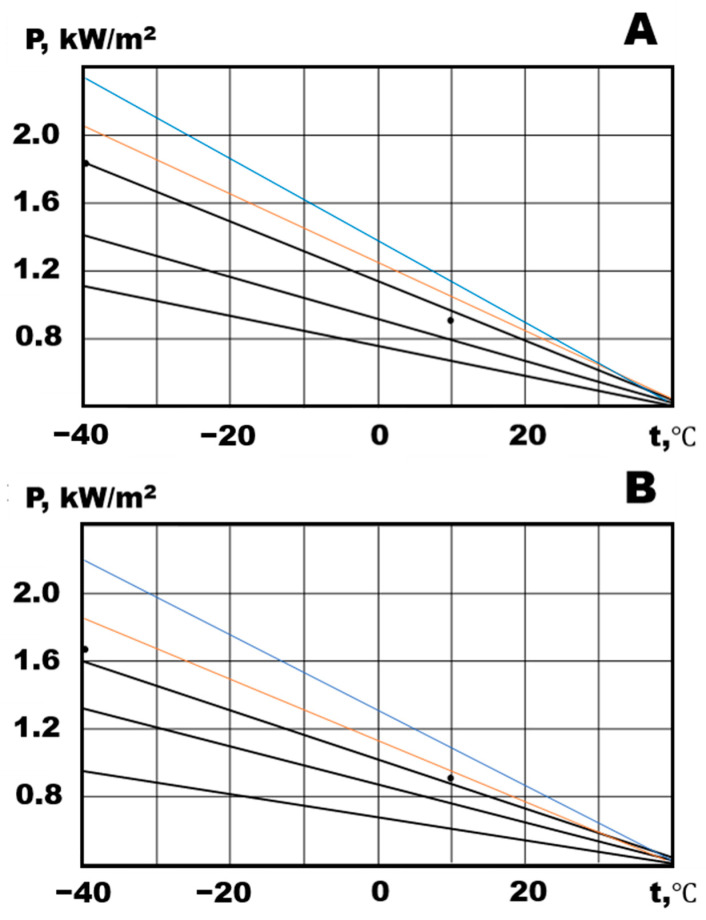
Specific power of heat: (**A**) NCO 1 and (**B**) NCO 2.

It is of interest to consider the change in the specific power of heat release. Degradation processes in the conductive structures of the composite lead to a decrease in it (Figure 6).

**Figure 7:**
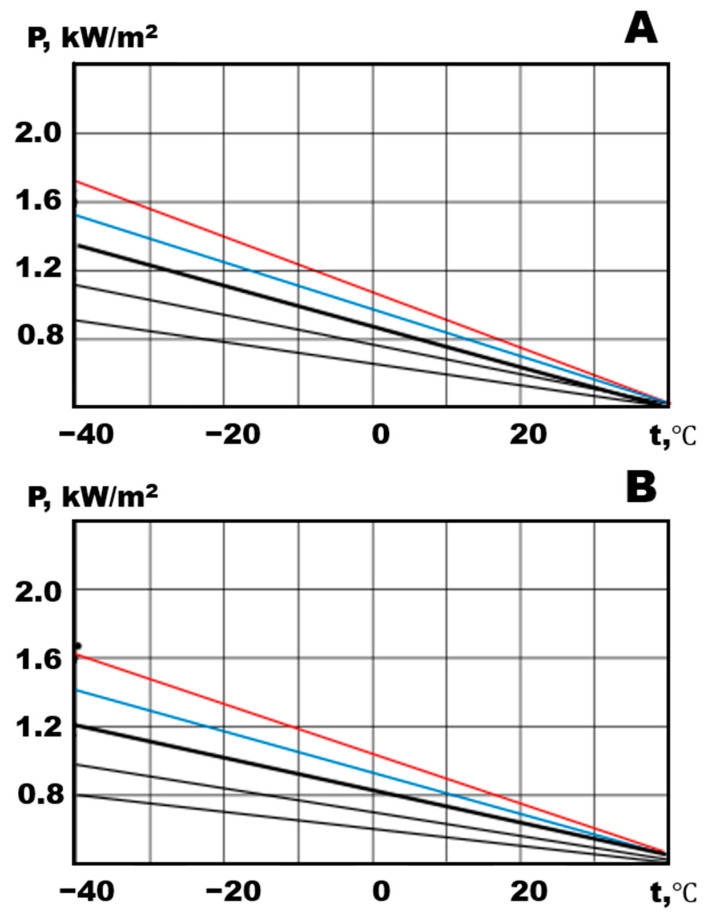
Specific power of heat: (**A**) NPU 1 and (**B**) NPU 2.

Let us consider the change in the specific power of heat release in the case of heaters with a polyurethane matrix (Figure 7). A comparable concentration of MWCNTs for polyurethane composites in comparison with organic silicon provides a lower value of the power level. Degradation processes in the conductive structures of the composite lead to a decrease in the specific power for both NPU 1 and NPU2. However, when first turned on, there is a power surge leading to an increase in power (red and blue lines, both for NPU 1 and NPU2). This observation is consistent with the thesis of the pulsed destruction of conducting structures based on carbon nanotubes in a polymer matrix [33].

## 4. Discussion

Studies of elastomers modified with MWCNTs were carried out at MWCNTs concentrations of 3 and 10 wt.% (Ni/MgO and Co-Mo/MgO). The modes of heat release of nanomodified elastomers at voltages of 220, 250, and 300 V demonstrate some decrements of heat release for composites with MWCNTs based on Ni/MgO and Co-Mo/MgO. The presented studies make it possible to take the next step in the development of functional materials for electric heating and the level of variation of the supply voltage, which will allow us to obtain information about the physicochemical parameters, following the principles of measuring the heat release that occurs during the transition of electrical energy into thermal energy.

In parallel with infrared thermography, changes in the dependence of specific power on ambient temperature conditions were demonstrated. These data are useful for the development of reliable and energy-efficient functional materials for electric heating based on elastic matrices and nanosized conductive fillers.

The influence of the polymer matrix on the power degradation process was established, from which it follows that, when using a polyurethane matrix, the degradation process is lower by 2.59% for MWCNTs based on Ni/MgO and 10.42% for MWCNTs based on Co-Mo/MgO; however, at the same time, the power of heat release at comparable concentrations of conductive filler (10 and 3 wt.% (Ni/MgO and Co-Mo/MgO)) is lower. From the point of view of the nature of the polymer, this can be explained by the better thermal stability of polyurethane, since local overheating at the point of contact or adhesion of the polymer and conductive structures is more effective with thermal stability. The initially higher heat release rate for the organic silicon compound can be explained by better adhesion in the structure of the CNT polymer.

The developed heaters can be used in the technology for the electric heating of car interiors [41]. Comparison of the obtained heaters (NCO 1/NCO 2; NPU 1/NPU 2) with existing analogs [42,43,44,45,46,47,48,49,50] in Table 2 leads to a conclusion about those that are the most adaptable to the supply voltage of 220 V, which makes it possible to implement more economical heating modes in household appliances, such as thermal fans and heaters. The conducted studies have shown the safety of using heaters at a higher voltage of up to 300 V, which does not lead to their ignition but only causes degradation of electrophysical parameters without loss of performance.

## 5. Conclusions

Let us conclude with the consequences of the observed changes in the electrical parameters of MWCNTs-containing elastomer samples. It has been established that an increase in voltage relative to the nominal 220 V by 13.64% leads to an initial peak power value with an increase of 16.67% and a decrease in power (for a temperature of −40 °C) by 22.22%, and by 36.36% (80 V)—a 33.33% increase in peak and a 38.89% derating for Ni/MgO-based MWCNTs and 18.75% to 43.75% (initial increase and subsequent decrease) for MWCNT based on Co-Mo/MgO with steady-state conditions after degradation is 18.75% and 43.75% less than nominal.

As for a polyurethane matrix, an increase in voltage relative to the nominal 220 V by 13.64% leads to an initial peak power value with an increase of 15.38% and a decrease in power (for a temperature of −40 °C) by 15.38% and by 36% (80 V)—a 30.77% increase in the peak and a 30.77% derating for Ni/MgO-based MWCNTs and from 16.67% to 33.33% (initial increase and subsequent decrease) for MWCNTs based on Co-Mo/MgO with steady-state conditions after degradation from 16.67% to 33.33% less than the nominal value.

The influence of the polymer matrix on the process of power-caused structural degradation was established, from which it follows that, when using a polyurethane matrix, the degradation process is lower (by 2.59% for MWCNTs based on Ni/MgO and 10.42% for MWCNTs based on Co-Mo/MgO), but at the same time, the power of heat release at comparable concentrations of conductive filler (10 and 3 wt.% (Ni/MgO and Co-Mo/MgO)) is lower.

In our upcoming studies, we are going to develop technologies based on these results.

## Figures and Tables

**Table 1:** Elastomers modified with MWCNT.

MWCNTs Catalyst for Synthesis	Elastomer’s Designation	
	CO (Silicon-Organic Compound)	PU (Polyurethane)
Ni/MgO	NCO 1	NPU 1
Co-Mo/MgO	NCO 2	NPU 2

**Table 2:** Comparing the electrothermal properties of different materials.

##	Materials	Voltage, V	Area, cm^2^	Temperature °C
1	CCSCF [42]	10	2 × 2	105
2	rGo/PET [43]	10	2 × 4	73
3	CNT [44]	20	2 × 2	90
4	rGO/CNT/NR [45]	15	-	69.1
5	CNT/PU [46]	2,5	-	70.4
6	CNT-embedded electric heating composites [47]	20	-	80
7	carbon fiber (CF)/asphalt mastics [48]	60	-	5
78	silver nanowire (AgNW) microgrid (AMG) structures [49]	2	-	51.4
79	F-N Co-Doped Graphene Oxide with Extended Sp2 Networks [50]	9	-	365
10	Under investigation: NCO 1/NCO 2 NPU 1/NPU 2	220	10 × 7	64.2/57.3 58.3/55.2

## Data Availability

The data presented in this study are available on request from the corresponding author.
